# The Common Prescription Patterns Based on the Hierarchical Clustering of Herb-Pairs Efficacies

**DOI:** 10.1155/2016/6373270

**Published:** 2016-04-10

**Authors:** Jia Cao

**Affiliations:** Department of Information Technology, Beijing Forestry University, Beijing 100083, China

## Abstract

Prescription patterns are rules or regularities used to generate, recognize, or judge a prescription. Most of existing studies focused on the specific prescription patterns for diverse diseases or syndromes, while little attention was paid to the common patterns, which reflect the global view of the regularities of prescriptions. In this paper, we designed a method CPPM to find the common prescription patterns. The CPPM is based on the hierarchical clustering of herb-pair efficacies (HPEs). Firstly, HPEs were hierarchically clustered; secondly, the individual herbs are labeled by the HPE*C* (the clusters of HPEs); and then the prescription patterns were extracted from the combinations of HPE*C*; finally the common patterns are recognized statistically. The results showed that HPEs have hierarchical clustering structure. When the clustering level is 2 and the HPEs were classified into two clusters, the common prescription patterns are obvious. Among 332 candidate prescriptions, 319 prescriptions follow the common patterns. The description of the patterns is that if a prescription contains the herbs of the cluster (*C*_1_), it is very likely to have other herbs of another cluster (*C*_2_); while a prescription has the herbs of *C*_2_, it may have no herbs of *C*_1_. Finally, we discussed that the common patterns are mathematically coincident with the Blood-Qi theory.

## 1. Introduction

The processes of diagnosing syndrome and prescribing prescriptions in TCM (traditional Chinese medicine) are empirical. It is essential to minimize the uncertainty caused by human factors by finding the unchangeable TCM patterns, syndrome pattern, and prescriptions pattern.

As far as the first kind of TCM patterns, syndrome pattern, is concerned, several syndrome patterns had been proposed, such as SEM (structure equation modeling) to explore the diagnosis of the suboptimal health status [1] and some syndrome diagnostic models for chronic gastritis [2] or for tuberculosis [3].

Here we studied the second kind of TCM patterns, prescription patterns, which are rules or regularities used to generate, recognize, or judge prescriptions. In TCM, the herbs in one prescription are not organized randomly, but according to a set of principles for the therapeutic purposes of mutual enhancement, mutual assistance, mutual restraint, or mutual antagonism [4]. The prescription patterns would reflect some of the principles in a formalized way.

Before discussing the prescription patterns in further detail, the TCM data relationships used in this paper were described firstly. There are three forms of TCM data, individual herbs (herbs), herb-pairs, and prescriptions. An herb-pair is composed of two herbs for the purposes of mutual enhancement, mutual assistance, mutual restraint, or mutual antagonism [5]; a prescription is composed of several herbs. Each herb has some TCM defined properties, including five fundamental natures (cold, cool, neutral, etc.), seven flavors (sour, bitter, sweet, etc.), and twelve meridians (liver, heart, spleen, etc.), and these properties have been standardized by the Chinese government in 2005 [6] and in 2010 [7]. Each herb-pair is associated with some* herb-pairs efficacies* (HPEs) which are also a kind of TCM defined properties.

The existing prescription patterns vary from methods to methods. By clustering algorithms, the patterns are in the form of specific groups of herbs for stroke [8] or for gout and hyperuricemia [9], in the form of latent tree for “disharmony between liver and spleen” which is a TCM defined symptom [10], in the form of several flat groups of herbs [4, 11, 12] with different treatment functions. By genetic algorithms, the patterns are core groups of herbs for lung cancer [12]. By factor analysis, the patterns are 7 groups of herbs for insomnia [13]. By association analysis, the patterns are the combinations of the herbal properties varied with different treatment purposes [14] and some herb combinations for psoriasis vulgaris [15]. Except for the patterns composed of properties of individual herbs [14], most of prescription patterns are core herbs for a certain disease or syndrome.

Up to now, too much attention has been paid to the specific patterns for diverse diseases or syndromes, while little attention is paid to the common patterns of all prescriptions. A particular pattern is suitable to generate or recognize a specific prescription for a certain disease, while the common patterns are also important when judging the feasibility of a prescription at a high overall level. Common features of complex systems are ubiquitous, such as small world [16] and scale-free [17] in social, biological, and information systems [18]. Usually, the knowledge of a few entities of a complex system does not straightforwardly lead to a description of the overall system [19]. The prescription of TCM as a complex system and the specific pattern of a single disease cannot reflect the global view of the regularities of prescriptions. So it is necessary to explore the common prescription patterns.

To explore the common prescription patterns, there are some questions: are prescriptions characterized by any common features? Is it possible to extract some mathematical expressions of the common features for all prescriptions? We discussed the possibility of solving the problems from two aspects.

*(1) Methodology*. The prescriptions are one kind of complex systems. In fact, most of complex systems have nested hierarchical structure; that is, the elements of the system can be partitioned into cluster which in turn can be partitioned into subclusters and so on up to a certain level. In biological taxonomy, individuals are grouped into species, species into genera, genera into families, and so on. Hierarchical clustering structure reflects both difference and common feature of the complex systems. The clustering at the top of the hierarchical tree is 1-clustering where all elements are in one cluster; the clustering at the bottom is element-clustering where every element is in different clusters. The higher clustering reflects commonality and the lower clustering reflects diversity. If the prescription-related properties show the hierarchical clustering structure, the herbal relations in the prescriptions would be represented by hierarchical clustering structure. Consequently, the commonality of the prescription patterns could be explored by higher clustering. The hierarchical clustering methods bring us hierarchical representations, rather than flat ones [20]. 

*(2) Data Foundation*. More than 100,000 herbal prescription records were accumulated from 180 BC to 1904 [21]. There are plenty of data to support statistical analysis on the prescription patterns. On the other hand, the prescription patterns would be extracted from the latent relations in the herb-pair data. The herb-pair based method is one of six methods of selecting herbs for a prescription [22], where several herb-pairs are selected to form a prescription [23]. So there must be some relationships between herb-pairs and prescriptions.

In this paper, we dug the common prescription patterns from the herb-pair data by hierarchical clustering methods and summarized a mathematic expression of the common patterns.

## 2. Material and Methods

### 2.1. Data Description

An herb-pair is composed of two herbs and provides some synergistic efficacy in vivo. In this study, 697 herb-pairs were directly collected from two reputable TCM literatures [24, 25]. One literature [25] had been printed four times and used as the data source in the research on herb-pairs [5]; another book [24] was supported by Shandong Academy of Chinese Medicine, Shandong University of Traditional Chinese Medicine, and its affiliated hospitals, and it is a reference book for the TCM graduate students. The data in both books were the TCM clinical records and every herb-pair and its efficacy were recorded in explicit form. Thus, 697 herb-pairs with 376 herbs and 32 HPEs were collected, and all HPEs were listed in Table 1.

332 prescriptions were collected from two popular books:* Treatise on Febrile Diseases* [26] and a collection [21]. The first book is a classical ancient book in TCM. The latter one is a popular current textbook in TCM High Education School, which was one of the series textbooks supported by State Administration of TCM of People's Republic of China. The prescriptions are frequently practiced and have been verified in the clinical TCM practices.

The symbols used in this paper are as follows. Let *H* = {*h*_1_, *h*_2_, *h*_3_⋯} be the set of individual herbs. Let *E* = {*e*_1_, *e*_2_, *e*_3_ ⋯ *e*_32_} be the set of HPEs. An herb-pair of *h*_*i*_ and *h*_*j*_ with *e*_*x*_ efficacy is denoted by a triplet (*h*_*i*_, *h*_*j*_, *e*_*x*_), where *e*_*x*_ belongs to *E*. Let *P* be the set of prescriptions, *P* = {*p*_1_, *p*_2_, *p*_3_ ⋯ *p*_*N*_}, where *p*_*i*_ is a prescription. Each prescription is composed of a set of herbs, *p*_*i*_ = {*h*_*i*_∣*h*_*i*_ ∈ *H*}.

### 2.2. Similarity Matrix of HPEs

Jaccard similarity coefficient is a statistic metric used for comparing the similarity and diversity of finite sample sets. It is defined as the size of the intersection divided by the size of the union of the sample sets. Given two sets *A* and *B*, their Jaccard similarity coefficient is

(1) $$\begin{array}{c} J_{\left(A,B\right)}=\frac{\left|A\cap B\right|}{\left|A\cup B\right|}, \end{array}$$

and 0 ≤ *J*_(*A*,*B*)_ ≤ 1. The larger the value is, the more similar the two sets are. Obviously, *J*_(*A*,*B*)_ = *J*_(*B*,*A*)_ and *J*_(*A*,*A*)_ = 1.

Jaccard coefficient was used to compare the similarity of any two HPEs. An HPE is denoted by a set of individual herbs which make up the herb-pairs with the HPE. The number of HPEs is 32, and the similarities between these efficacies can be represented as a similarity matrix, *J*_32×32_.

For example, given a number of herb-pairs which are

(2) $$\begin{array}{c} \left(\;a,b,e_{x}\;\right),\\ \left(\;b,d,e_{x}\;\right),\\ \left(\;c,d,e_{x}\;\right),\\ \left(\;d,f,e_{y}\;\right), \end{array}$$

where *e*_*x*_ ∈ *E*, *e*_*y*_ ∈ *E*, the herbal set of *e*_*x*_ is {*a*, *b*, *c*, *d*}, and the set of *e*_*y*_ are {*d*, *f*}. The Jaccard coefficient between *e*_*x*_ and *e*_*y*_ is *J*_(*x*,*y*)_ = 0.2. The HPE similarity matrix of this example is

(3) $$\begin{array}{c} \left[\begin{array}{cc} 1 & 0.2\\ 0.2 & 1 \end{array}\right]. \end{array}$$

### 2.3. Solving the Problem

In this section, a method of finding the common prescription patterns (CPPM) was proposed. The basic idea of CPPM was to use the clusters of HPEs to represent the common patterns of prescriptions. The methodological possibility had been elaborated in Section 1.

#### 2.3.1. An Example: Extracting the Patterns at Different Levels of HPE Hierarchical Clustering

Hierarchical clustering structure reflects both individual and common features of complex systems. The hierarchical clustering of HPEs could be used to study the individual problem or the common problem. The higher clustering of HPEs reflects commonality and the lower clustering reflects individuality.

Here is an example to show how to extract the common patterns and the specific patterns of prescriptions at the different levels of the HPE hierarchical clustering.

**Figure 1:**
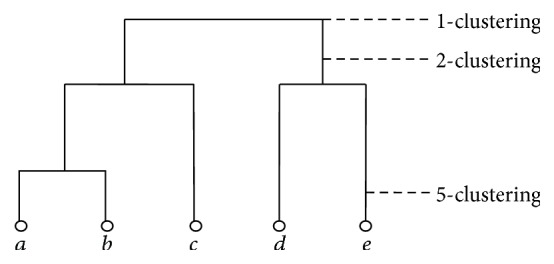
The hierarchical clustering structure of the five HPEs.

*(1) Hierarchical Clustering Structure of HPEs*. Given HPEs, *a*, *b*, *c*, *d*, and *e*, the hierarchical clustering structure of the HPEs specifies clustering at all granularities, shown in Figure 1. The 1-clustering is *C*_1_ = {*a*, *b*, *c*, *d*, *e*}, the 2-clustering is *C*_1_ = {*a*, *b*, *c*}, *C*_2_ = {*d*, *e*}, and the 5-clustering is *C*_1_ = {*a*}, *C*_2_ = {*b*}, *C*_3_ = {*c*}, *C*_4_ = {*d*}, and *C*_5_ = {*e*}. The 5-clustering at the bottom is element-clustering where different HPE belongs to different clusters. The *r*-clustering is *r*-level of the hierarchical tree. 

*(2) Extracting the Patterns at Different r-Levels of HPEs.* Given 5 prescriptions and their patterns of 5-clustering listed in Table 2, the other patterns at different clustering levels of HPEs were listed in Table 2. It is easy to recognize the common patterns at the 2-clustering and 1-clustering; it is also easy to recognize the core-set at the 5-clustering. The patterns of 5-clustering are diverse that every prescription has a different pattern. Most of existing researches on the TCM prescription patterns were conducted at this level. That is why these studies focus on recognition of the core herbs. Our study here was conducted at the higher level to seek the common patterns.

#### 2.3.2. CPPM

The inputs and outputs of CPPM were shown as follows.

Inputs are as follows:

(i) Similarity matrix of HPEs.
(ii) Candidate prescriptions = {*p*_1_, *p*_2_, *p*_3_ ⋯ *p*_*N*_}.
(iii) The granularity level of hierarchical clustering *r*.

Outputs are as follows:

(i) *r*-clustering that is HPE*C*_1_, HPE*C*_2_ ⋯ HPE*C*_*r*_.
(ii) Common prescription patterns:
   combination of HPE*C* and POs.
(iii) OO.

The metrics PO and OO were used to evaluate the support of the results, which would be stated in Section 2.5. The steps of CPPM are as follows.

**Step 1 (get clustering of HPEs).**

A hierarchical clustering algorithm, such as Ward algorithm or BIRCH algorithm, was applied on the similarity matrix of HPEs. Then the *r*-clustering of HPEs was obtained, where the HPEs were clustered into *r* groups/clusters. A cluster of HPE is denoted by HPE*C*. Finally each HPE belongs to a specific HPE*C*.

**Step 2 (label the herbs by HPE*C*).**

There are two substeps: an herb may make up some herb-pairs with different HPEs. (1) The frequent HPE is its dominating HPE. (2) According to the dominating HPE, the corresponding HPE*C* is assigned as a label to the herb. Thus, every herb has a HPE*C*.

**Step 3 (get prescription patterns).**

For a prescription, replace its herbs by the corresponding HPE*C*s and get a HPE*C* sequence of the prescription and then take the HPE*C* sequence without repeating as the pattern of the prescription. Some herbs of prescriptions cannot be located in the herb-pairs. If the input of CPPM is N prescriptions, N patterns would be obtained in this step.

**Step 4 (find the common patterns of prescriptions).**

The metric PO of each pattern is calculated and the frequent patterns with higher value of PO are selected as the common prescription patterns.

### 2.4. Levels of Granularity

By inputting different parameter *r* in the 1st step of CPPM, different levels of granularity of patterns can be output.

At the top level (*r* = 1), all HPEs belong to a common HPE*C* in 1st step, all herbs also belong to the HPE*C* in 2nd step, and the herbs of prescriptions will be projected to the common HPE*C* in 3rd step. The common HPE*C* is the common pattern, but it is obviously meaningless. At the bottom level (*r* = |*H*|), every HPE belongs to different HPE*C*s and the herbs will be projected to all HPEs in 3rd step. Consequently, the prescription patterns are numerous, and it is hard to find the common prescription patterns in 4th step.

It is not easy to determine the level of granularity. A good granularity level should be a meaningful clustering, which should be coincident with a certain biological action of mechanism or a TCM theory. Here we performed the process in the manual way which would be stated in Section 4.

### 2.5. Evaluating Metrics

#### 2.5.1. Overlap Coefficient

There are two different datasets with different data sources in CPPM, herb-pairs and prescriptions. Through the mapping technique, herb-pairs and prescriptions established a certain connection. The size of the intersection of the two sets reflects their consistency and completeness. Let |*X*| be the size of the set *X*. Let *H*_herb-pair_ be the herbs without repeating of herb-pairs; let *H*_prescription_ be the herbs without repeating of the candidate prescriptions. The overlap coefficient (OO) is to evaluate the consistency and completeness of the two sets. It is better to get a large value of OO. Consider

(4) $$\begin{array}{c} OO_{\text{without-repeating}}=\frac{\left|H_{prescription}\cap H_{herb\text{-}pair}\right|}{\min\left\{\left|H_{prescription}\right|,\left|H_{\text{herb-pair}}\right|\right\}}. \end{array}$$

#### 2.5.2. Probability of Occurrence

To identify the commonality of the prescription patterns, we designed the probability of occurrence (PO). Let *F*_pattern_ be the number of prescriptions with a certain pattern and |*P*| the number of all inputted prescriptions. The PO of a certain pattern is defined as

(5) $$\begin{array}{c} PO_{\text{pattern}}=\frac{F_{\text{pattern}}}{\left|P\right|}. \end{array}$$

## 3. Results

### 3.1. Herb-Pair Efficacy Size

The size of an herb-pair efficacy is the number of herb-pairs with the herb-pair efficacy. The herb-pair efficacy sizes of different efficacies are not the same (in Table 1). The efficacy of *e*_23_ has quite large number of herb-pairs, while few herb-pairs provide *e*_13_ and *e*_29_. The smaller herb-pairs efficacies are unsuitable for statistical analysis, so we delete the smaller herb-pairs efficacies, whose size is smaller than 12. 12 is the threshold value because *e*_18_ is one of the specific observed objects and the reason was in Section 4. Our previous study results showed that *e*_1_ and *e*_11_ are two common herb-pairs efficacies [27] and should be omitted too. Consequently, the candidate herb-pairs efficacies were marked with “√” in Table 1, which are *e*_2_, *e*_3_, *e*_5_, *e*_6_, *e*_7_, *e*_8_, *e*_9_, *e*_10_, *e*_16_, *e*_17_, *e*_18_, *e*_19_, *e*_20_, *e*_21_, *e*_22_, *e*_23_, *e*_24_, *e*_25_, *e*_26_, *e*_30_, and *e*_32_.

### 3.2. Classification of HPEs

**Figure 2:**
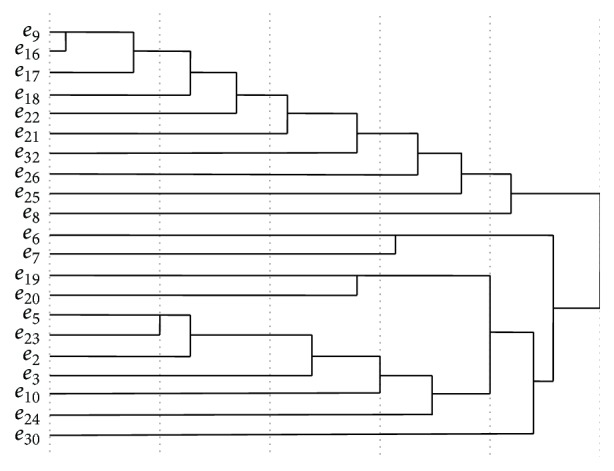
The hierarchical tree of HPEs, where the nodes of left are the IDs of HPEs.

A hierarchical clustering method (average linkage within groups) applied to *J*_32×32_. The hierarchical tree of HPEs was shown in Figure 2. If the level of granularity is 2, the 2-clustering is {HPE*C*_1_, HPE*C*_2_}, where

(6) HPEC1=e2,e3,e5,e6,e7,e10,e19,e20,e23,e24,e30,HPEC2=e8,e9,e16,e17,e18,e21,e22,e25,e26,e32.

The clusters at 2-level were listed in Table 1. Because the main goal of this paper is to find the common patterns, some significant results were shown when the clustering level is 2. So the situation of 2-clustering was discussed here.

### 3.3. Classification of Herbs

Here we counted the number of herb-pairs whose two herbs belong to one cluster, HPE*C*_1_ or HPE*C*_2_. The detailed results were listed in Table 1. As we can see, there are very few exceptional cases that two herbs are in one cluster, while the herb-pairs composed of the two herbs maybe provide the efficacy of another cluster. So the statistic shows the 2-clustering at the level of HPE is consistent at level of individual herbs. Thus the 3rd step is validated.

### 3.4. Overlap Coefficient

The number of herbs in the sets HPE*C*_1_ and HPE*C*_2_ that are candidate herbs at the herb-pairs level is |*H*_herb-pair_| = 330. There are 332 prescriptions composed of 320 herbs, |*H*_prescription_| = 320. The size of the intersection of herbs in both herb-pairs and prescriptions is |*H*_herb-pair_∩*H*_prescription_| = 192. So

(7) $$\begin{array}{c} OO=0.6. \end{array}$$

The number of all herbs in all the inputted prescriptions with repeating is 2057, where 1677 herbs could be located in *H*_herb-pair_. The value of overlap coefficient is about 0.81.

### 3.5. Prescription Patterns

To get the prescription patterns, we projected the herbs of the prescriptions to the two clusters, HPE*C*_1_ and HPE*C*_2_. The maximum number of combinations of the two clusters is four. All four patterns, the number of prescriptions of each pattern, and corresponding PO were listed in Table 3.

### 3.6. Common Prescription Patterns

From the value of PO of each pattern, two common prescription patterns were recognized:

 Pattern 1: HPE*C*_1_ and HPE*C*_2_.
 Pattern 2: HPE*C*_1_.

The verbal description of the common patterns is as follows: if a prescription contains the herbs of HPE*C*_2_, it is very likely to have other herbs of HPE*C*_1_, while if a prescription has the herbs of HPE*C*_1_, it may have no herbs of HPE*C*_2_. It seems that the herbs in the HPE*C*_1_ are master and the herbs in the HPE*C*_2_ are auxiliary. The formalization of the common patterns is f.1. Consider

(f.1) $$\begin{array}{c} \text{in}\;\;HPEC_{1}\begin{array}{c} \overset{\text{always}}{\leftarrow }\\ \overset{\text{not}\;\;\text{always}}{\to } \end{array}\text{in}\;\;HPEC_{2}. \end{array}$$

## 4. Discussion

This section is quite argued and mysterious. But, for the sake of perfect mathematical matching, we found that the common prescription patterns are completely consistent with the Blood-Qi theory in TCM.

The Blood-Regulating efficacies, *e*_19_ and *e*_20_, are two of the therapeutic efficacies of HPE*C*_1_ and the Qi-Regulating efficacies, *e*_16_, *e*_17_, and *e*_18_, are the efficacies of HPE*C*_2_. So f.1 is equal to f.2. Consider

(f.2) $$\begin{array}{c} \text{Blood-Regulation}\begin{array}{c} \overset{\text{always}}{\leftarrow }\\ \overset{\text{not always}}{\to } \end{array}\text{Qi-Regulation}. \end{array}$$

In the Blood-Qi theory of TCM, Qi stagnation leads to blood stasis and blood stasis does not always cause Qi stagnation. So the formalization of the theory is f.3. Consider

(f.3) $$\begin{array}{c} \text{Blood stasis}\begin{array}{c} \overset{\text{always}}{\leftarrow }\\ \overset{\text{not always}}{\to } \end{array}\text{Qi stagnation}. \end{array}$$

The two formulas f.1 and f.3 have completely the same form. So the common prescription patterns are coincident with the Blood-Qi theory in TCM.

## 5. Conclusions

The prescription of TCM is a complex system. Usually, the knowledge of a few entities (prescriptions) of a complex system does not straightforwardly lead to a description of the overall system. So the common prescription patterns can provide us with a global view of the regularities of prescriptions. A method CPPM proposed in this paper is to find the common prescription patterns based on the hierarchical clustering of herb-pairs efficacies. The method was applied on the 697 herb-pairs and the 332 prescriptions. The statistic results showed that when the granularity level of the hierarchical clustering is 2, the common patterns are obvious. The description of the common patterns is that if a prescription contains the herbs of the clusters (HPE*C*_1_) it is very likely to have other herbs of another cluster (HPE*C*_2_); while a prescription has the herbs of HPE*C*_2_, it may have no herbs of HPE*C*_1_. And the formalizations of the common patterns and the Blood-Qi theory showed mathematical consistency. With the common pattern information, if the herbs of a new unknown prescription do not follow the pattern, the prescription is incorrect and inappropriate with a very large probability. Thus the common pattern reflects a kind of prescription regularity and can also be used to judge the appropriateness of a new prescription at a high level.

## Figures and Tables

**Table 1:** HPEs and some statistics.

ID	Efficacy	Size	Candidate	HPE*C* in 2-clustering	The number of herb-pairs composed of herbs in HPE*C*_1_ and HPE*C*_2_	
					HPE*C*_1_	HPE*C*_2_
*e*_1_	Expelling wind and dispersing cold	35				
*e*_2_	Dispelling wind and clearing hot	24	√	HPE*C*_1_	20	0
*e*_3_	Warming Zang-fu organs	17	√	HPE*C*_1_	17	0
*e*_4_	Warming meridians	9				
*e*_5_	Clearing hot and purging fire	46	√	HPE*C*_1_	42	1
*e*_6_	Clearing hot and cooling blood	17	√	HPE*C*_1_	17	0
*e*_7_	Clearing hot and detoxicating	19	√	HPE*C*_1_	17	2
*e*_8_	Clearing deficient hot	12	√	HPE*C*_2_	4	7
*e*_9_	Resolving and drying dampness	19	√	HPE*C*_2_	5	14
*e*_10_	Promoting urination and dehumidification	34	√	HPE*C*_1_	31	1
*e*_11_	Clearing wind and damp	14				
*e*_12_	Cold purgation	6				
*e*_13_	Warm purgation	4				
*e*_14_	Moistened cathartic	5				
*e*_15_	Dispelling retained water	7				
*e*_16_	Regulating Qi	24	√	HPE*C*_2_	2	20
*e*_17_	Promoting Qi	18	√	HPE*C*_2_	3	15
*e*_18_	Depressing Qi	12	√	HPE*C*_2_	0	12
*e*_19_	Activating blood	40	√	HPE*C*_1_	37	2
*e*_20_	Hemostasis	26	√	HPE*C*_1_	26	0
*e*_21_	Relieving cough and asthma	33	√	HPE*C*_2_	7	22
*e*_22_	Elimination	22	√	HPE*C*_2_	4	18
*e*_23_	Supplying Qi and blood	135	√	HPE*C*_1_	131	1
*e*_24_	Astringing	29	√	HPE*C*_1_	28	1
*e*_25_	Extinguishing wind	21	√	HPE*C*_2_	3	18
*e*_26_	Tranquilization	16	√	HPE*C*_2_	3	13
*e*_27_	Resuscitation	11				
*e*_28_	Expelling parasite	4				
*e*_29_	Emetics	3				
*e*_30_	External application	16	√	HPE*C*_1_	15	1
*e*_31_	Clearing hot and drying dampness	6				
*e*_32_	Eliminating phlegm	13	√	HPE*C*_2_	0	12
Summary		697			412	160

**Table 2:** Prescription patterns at different levels of granularity.

Prescriptions	Prescription patterns		
	5-clustering	2-clustering	1-clustering
*p*_1_	*C*_1_, *C*_2_, *C*_3_, *C*_4_, *C*_5_	*C*_1_, *C*_2_	*C*_1_
*p*_2_	*C*_2_, *C*_3_, *C*_5_	*C*_1_, *C*_2_	*C*_1_
*p*_3_	*C*_2_, *C*_3_	*C*_1_	*C*_1_
*p*_4_	*C*_2_, *C*_4_	*C*_1_, *C*_2_	*C*_1_
*p*_5_	*C*_1_, *C*_2_, *C*_3_	*C*_1_	*C*_1_
Results	Core-set: *C*_2_, *C*_3_	Common patterns: *C*_1_, *C*_2_ *C*_1_	Common pattern: *C*_1_

**Table 3:** Prescription patterns at level 2, 2-clustering of HPEs.

Four patterns	Number of prescriptions	PO
HPE*C*_1_, HPE*C*_2_	209	0.63
HPE*C*_1_	110	0.33
HPE*C*_2_	8	0.02
Other	5	0.02
Total	**332**	
